# Sex Steroids and Osteoarthritis: A Mendelian Randomization Study

**DOI:** 10.3389/fendo.2021.683226

**Published:** 2021-06-23

**Authors:** Yi-Shang Yan, Zihao Qu, Dan-Qing Yu, Wei Wang, Shigui Yan, He-Feng Huang

**Affiliations:** ^1^ The Key Laboratory of Reproductive Genetics (Zhejiang University), Ministry of Education, Zhejiang University School of Medicine, Hangzhou, China; ^2^ Department of Orthopedic Surgery, The Second Affiliated Hospital, Zhejiang University School of Medicine, Hangzhou, China; ^3^ Department of Osteology, Orthopedic Research Institute of Zhejiang University, Hangzhou, China; ^4^ The Second Affiliated Hospital, School of Medicine, Zhejiang University, Hangzhou, China

**Keywords:** Mendelian randomization, osteoarthritis, dehydroepiandrosterone sulfate, estradiol, testosterone, dihydrotestosterone

## Abstract

**Objective:**

Sex steroids are thought to contribute to the pathogenesis of osteoarthritis (OA). This study investigated the causal role of sex steroids in site- and sex-specific OA and risk of joint replacement surgery using the Mendelian randomization (MR) method.

**Methods:**

Instrumental variables for estradiol, dehydroepiandrosterone sulfate, testosterone (T), and dihydrotestosterone (DHT) were selected. We used the inverse variance weighting (IVW) approach as the main MR method to estimate causal effects based on the summary-level data for OA and joint replacement surgery from genome-wide association studies (GWAS).

**Results:**

A positive causal association was observed between serum T level and risks of hip OA (odds ratio [OR]=1.558, 95% confidence interval [CI]: 1.193–2.034; P=0.001) and hip replacement (OR=1.013, 95% CI: 1.008–1.018; P=2.15×10^−8^). Serum DHT level was also positively associated with the risk of hip replacement (OR=1.011, 95% CI: 1.006–1.015; P=4.03×10^−7^) and had potential causality with hip OA (OR=1.398, 95% CI: 1.054–1.855; P=0.020).

**Conclusions:**

Serum T and DHT levels may play causal roles in the development of hip OA and contribute to the risk of hip replacement, although the underlying mechanisms require further investigation.

## Introduction

Osteoarthritis (OA) is the most common degenerative joint disorder worldwide (1) and one of the main causes of years lived with disability according to the 2010 World Health Organization Global Burden of Disease study (2, 3). OA is clinically characterized by chronic pain, morning stiffness, and crepitus along with radiographic findings in diarthrodial joints such as the knee and hip. The pathogenesis of OA is not fully understood, but excessive physiologic activity and an overload of pathologic factors such as inflammatory cytokines (4) and matrix degradation (5) are known to contribute. Standard treatment for OA includes early prevention and pharmacotherapy, while joint replacement surgery (6) is effective for end-stage disease (7). Additionally, hormone replacement therapy (HRT) was shown to reduce the revision rate after total knee or hip arthroplasty by almost 40% (8). Middle-aged women are more likely than men to be affected by OA, especially in the hip or knee (9). Low plasma androstenedione concentration was shown to be associated with an increased risk of knee and hip arthroplasty in overweight men (10). The evidence to date indicates that sex steroids play an important role in the development of OA. However, a causal relationship between sex steroids and OA risk has not been established.

We recently reported a positive causal relationship between circulating sex hormone-binding globulin (SHBG) concentration (11) and calcium level (12) and risk of OA. Testosterone (T) and estradiol (E2) are active forms of sex steroids in males and females that are derived from inactive precursors such as dehydroepiandrosterone sulfate (DHEAS) and androstenedione in the circulation. E2 can also be formed from the aromatization of T, which can be converted to the more potent hormone dihydrotestosterone (DHT).

Genome-wide association studies (GWASs) have identified multiple genetic loci represented by single nucleotide polymorphisms (SNPs) that are closely associated with sex steroid concentrations (13). The Mendelian randomization (MR) (14) approach is widely used to evaluate the causal relationship between exposures and clinical outcomes based on summary data from GWASs with SNPs as instrumental variables. The fundamental principle of MR analysis is that genetic variants are randomly inherited at conception; because their distribution in a population is natural, it is presumed that the results of MR analyses are less susceptible to environmental influence and confounds. In the present study, we used validated SNPs and summary statistics from publicly available GWAS datasets to investigate the causal association between sex steroids and OA development with the MR method.

## Methods

### Selection of Instrumental Variables

E2, DHEAS, T, and DHT were selected as exposures. SNPs associated with each sex steroid were identified from GWAS datasets of European cohorts of the following sizes: E2, number(N)=11,907 (15); DHEAS, N=14,846 (16); T, N=3239 (17); and DHT, N=3239 (17). All SNPs selected as instrumental variables were correlated with the corresponding exposure at a genome-wide significance level (P<5×10^−8^). A linkage disequilibrium (LD) test (**Supplementary Table 1**) was performed on the LD-link website (https://ldlink.nci.nih.gov/; European, r^2^<0.2). Detailed information of the association between the selected SNPs and exposures is shown in **Table 1**. The potential confounders associated with the selected SNPs were analyzed in the PhenoScanner database(http://www.phenoscanner.medschl.cam.ac.uk/) (**Supplementary Table 2**).

**Table 1:** Characteristics of SNPs for exposures from GWAS.

Exposure	Gene	SNP	Chromosome: Position	EA	Association with exposure	
					β (SE)	P value
E_2_	CYP19A1	rs727479	15:51534547	A	1.39 (0.12)	8.2×10^-30^
E_2_	CYP19A1	rs16964258	15:51605408	G	2.13 (0.25)	8.2×10^-15^
E_2_	FAM9B	rs5934505	X:8913826	C	0.67 (0.12)	3.4×10^-8^
E_2_	MIR	rs5951794	X:146432188	G	0.68 (0.11)	3.1×10^-10^
DHEAS	BCL2L11	rs6738028	2:111949327	G	-0.04 (0.01)	1.72×10^-8^
DHEAS	ARPC1A	rs740160	7:98957880	T	0.15 (0.02)	1.56×10^-16^
DHEAS	TRIM4	rs17277546	7:99489571	A	-0.11 (0.02)	4.50×10^-11^
DHEAS	HHEX	rs2497306	10:94485211	C	-0.04 (0.01)	4.64×10^-9^
DHEAS	CYP2C9	rs2185570	10:96751270	C	-0.06 (0.01)	2.29×10^-8^
DHEAS	BMF	rs7181230	15:40360741	G	0.05 (0.01)	5.44×10^-11^
DHEAS	SULT2A1	rs2637125	19:48401893	A	-0.09 (0.01)	2.61×10^-19^
T	JMJD1C	rs10822186	10:65350383	A	-0.06 (0.01)	1.20×10^-8^
T	SHBG	rs4239258	17:7397043	A	-0.16 (0.03)	4.47×10^-8^
T	SHBG	rs34790908	17:7451110	T	0.07 (0.01)	1.66×10^-9^
T	SHBG	rs727428	17:7537792	T	-0.07 (0.01)	1.26×10^-12^
DHT	SHBG	rs4151121	17:7342294	G	0.10 (0.02)	7.96×10^-10^
DHT	SHBG	rs4265880	17:7396267	T	-0.24 (0.04)	1.89×10^-8^
DHT	SHBG	rs4227	17:7491177	G	0.09 (0.02)	3.68×10^-8^

Gene, nearest gene to the SNP; EA, effect allele; β, per allele effect on the exposure; SE, standard error; P value, p-value for the genetic association.

### Genetic Associations With Outcomes

Because both knee and hip are common sites of OA (3), summary data for overall OA and hip and knee OA were derived from a GWAS meta-analysis of UK Biobank and Arthritis Research UK Osteoarthritis Genetics datasets (18) that included 455,221; 393,873; and 403,124 European individuals. Given that sex is a risk factor of OA, summary-level data for OA in each sex was extracted from the UK Biobank (http://www.nealelab.is/uk-biobank), which included 30,046 cases of OA (19,397 women and 10,649 men) among 361,141 European subjects (194,153 women and 166,988 men). Summary data for hip and knee replacement surgery were also obtained from UK Medical Research Council Integrative Epidemiology Unit OpenGWAS Project (https://gwas.mrcieu.ac.uk/), which included 7322 cases of hip and 5657 cases of knee replacement among 462,933 European individuals. The association data of the selected instrumental variables and sex steroids along with outcomes are provided in **Supplementary Tables 3**–**5**. All studies contributing data to our analyses were approved by the relevant ethics committees, and all study participants provided written, informed consent (**Supplementary Table 6**).

### Statistical Analysis

A 2-sample MR approach was adopted in our study. Causal associations between sex steroids and risks of OA and joint replacement were estimated based on the random-effects inverse variance weighting (IVW) model (19). Since four separate outcomes are being tested in our study, the threshold for adjusted p-value was 0.0125. The analytical results with a p-value between 0.125 and 0.05 are considered nominally significant results.

A weighted median (WM) analysis, which involved calculating the median value of ratio instrumental variable estimates, was also performed as sensitivity analysis. The MR-Egger and MR pleiotropy residual sum and outlier (MR-PRESSO) (20) methods were used to account for pleiotropic effects (21) to exclude bias observed in the sensitivity analysis. Outlying genetic variants were identified by MR-PRESSO and used to correct the results. Moreover, SNPs associated with included outcomes (P<1×10^−4^) were removed from IVW in the sensitivity analysis. The estimated effects are reported as odds ratios (ORs) with 95% confidence intervals (CIs). We used R v3.6.1 and the R MendelianRandomization package (22) for all statistical analyses.

## Results

### Causal Associations Between Sex Steroid Levels and Overall and Site-Specific OA

**Figure 1:**
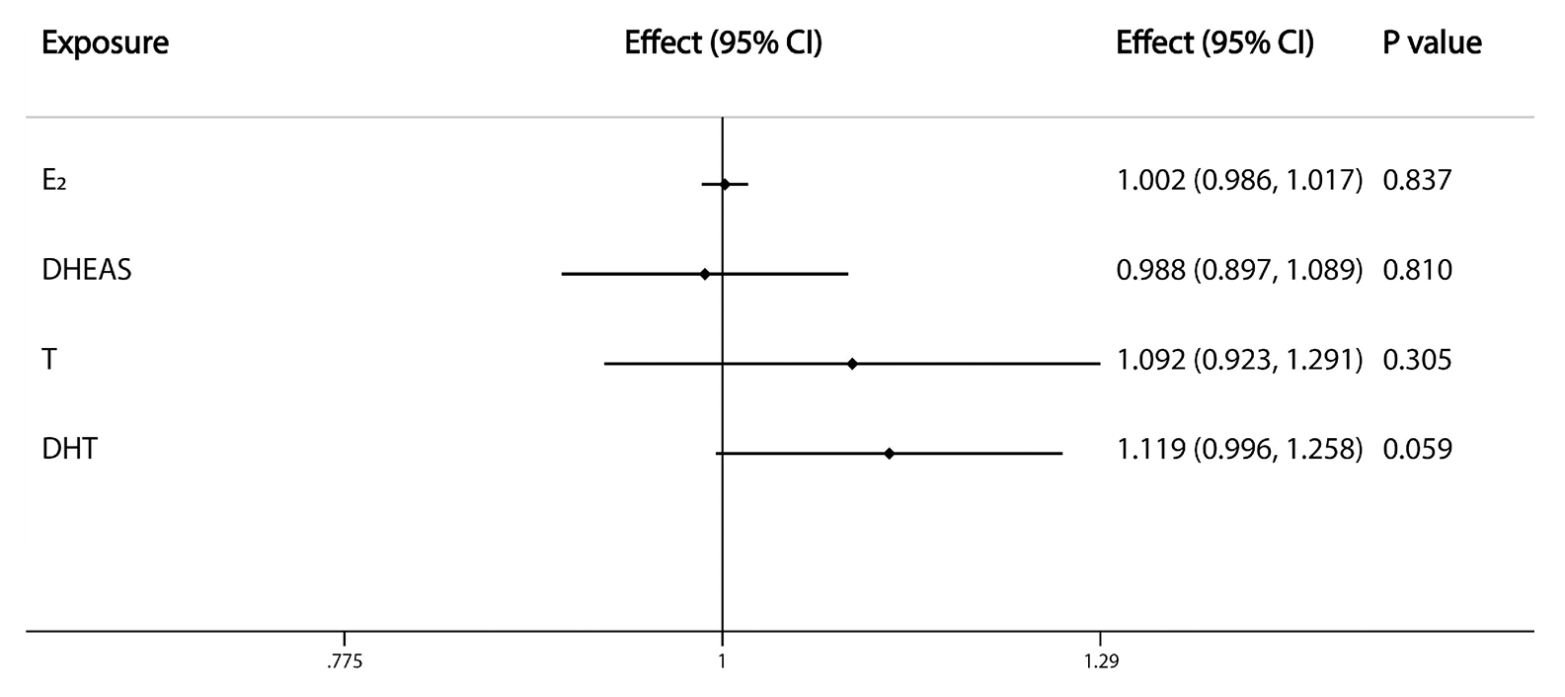
Causal effects of sex steroids on the risk of overall OA. The estimated effects, 95% confidence intervals and p-values of associations were contained. Effect, the combined causal effect; CI, confidence interval; P value, p-value of the causal estimate.

**Figure 2:**
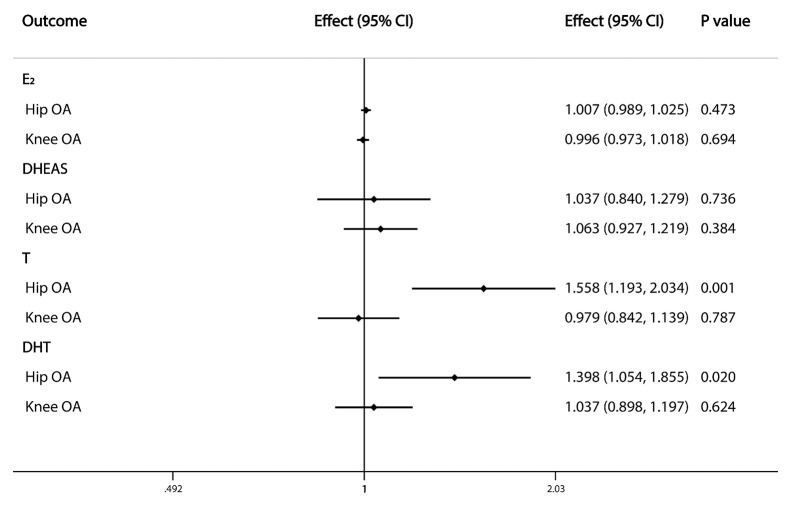
Causal effects of sex steroids on the risk of OA of hip and knee. The estimated effects, 95% confidence intervals and p-values of associations were contained. Effect, the combined causal effect; CI, confidence interval; P value, p-value of the causal estimate.

The primary IVW analyses provide no evidence for the causal relationship between the included sex steroids and overall OA (**Figure 1**). Nevertheless, the risk of hip OA was causally influenced by serum T levels (OR=1.558, 95% CI: 1.193–2.034; P=0.001). And the nominally significant results suggested a positive association between serum DHT levels (OR=1.398, 95% CI: 1.054–1.855; P=0.020) and hip OA. However, there was no evidence for associations between the levels of other sex steroids and hip and knee OA (**Figure 2**).

### Causal Association Between Sex Steroid Levels and OA by Sex

**Figure 3:**
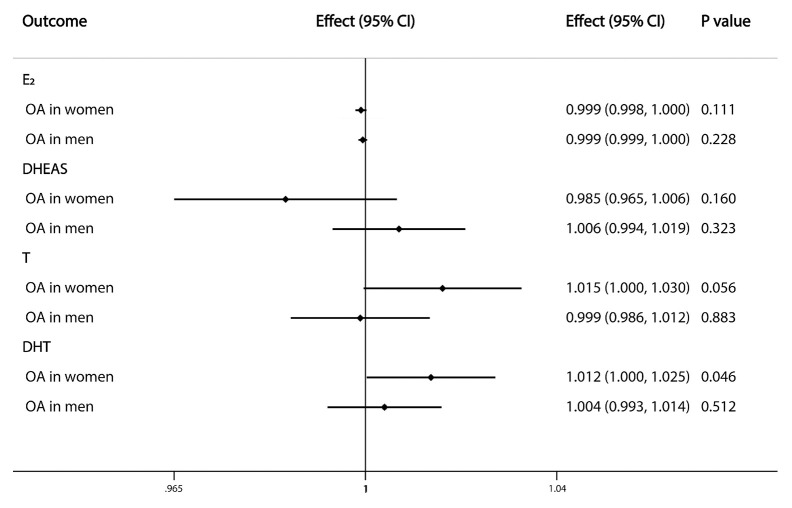
Causal effects of sex steroids on the risk of OA in single sex. The estimated effects, 95% confidence intervals and p-values of associations were contained. Effect, the combined causal effect; CI, confidence interval; P value, p-value of the causal estimate.

The nominally significant results of IVW analyses showed that serum DHT level tended to have a causal effect on the risk of OA in women (OR=1.012, 95% CI: 1.000–1.025; P=0.046), while no relationship was observed between sex steroid levels and OA risk in men (**Figure 3**).

### Causal Association Between Sex Steroid Levels and Joint Arthroplasty

**Figure 4:**
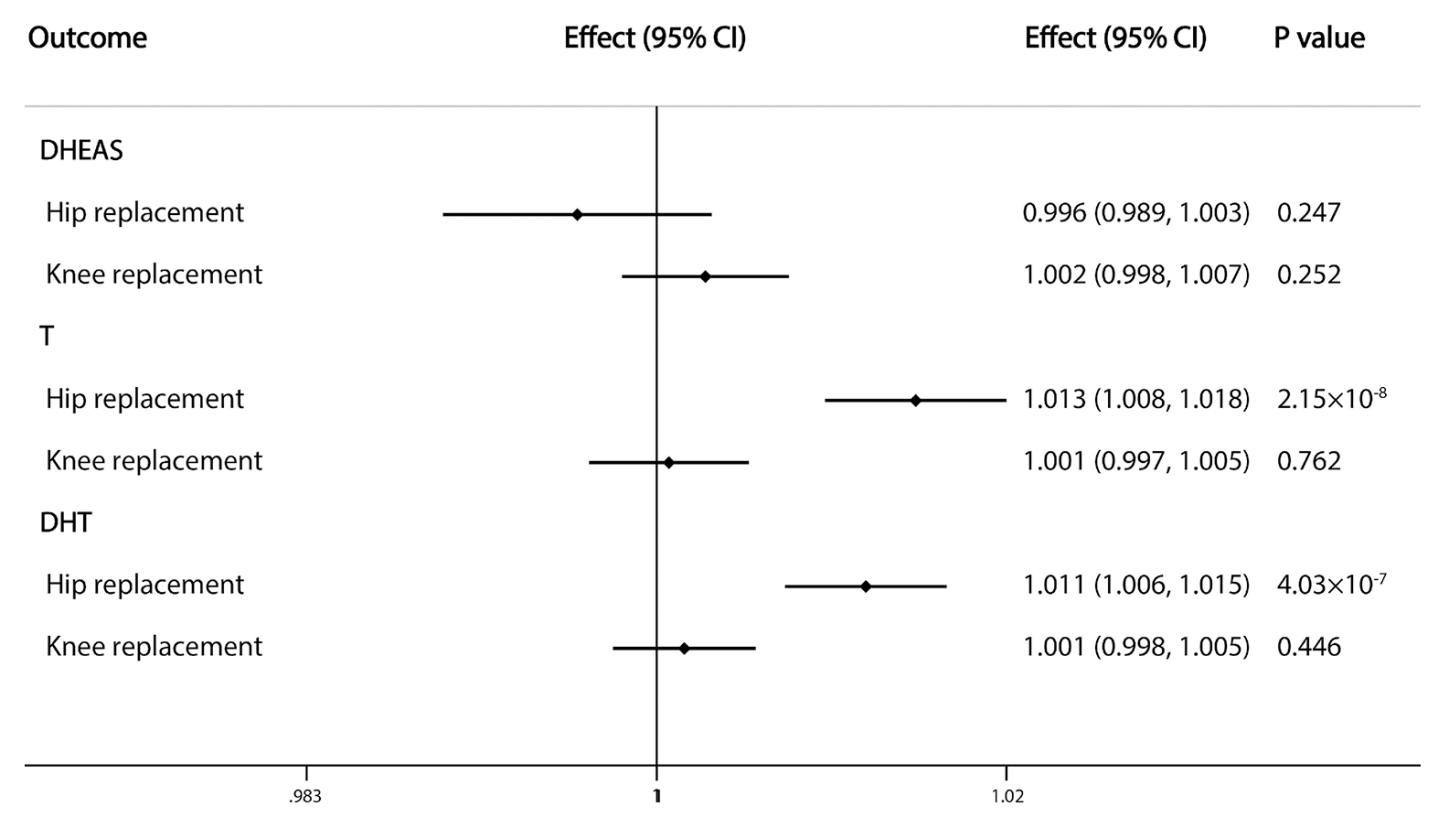
Causal effects of sex steroids on the risk of joint replacement of hip and knee. The estimated effects, 95% confidence intervals and p-values of associations were contained. Effect, the combined causal effect; CI, confidence interval; P value, p-value of the causal estimate.

Because of a lack of corresponding outcome data for E2-associated SNPs, the association between serum E2 level and joint replacement was not estimated by IVW analysis in our study. The results of the analysis showed that the risk of hip replacement was causally influenced by serum T (OR=1.013, 95% CI: 1.008–1.018; P=2.15×10^−8^) and DHT (OR=1.011, 95% CI: 1.006–1.015; P=4.03×10^−7^) levels. However, we found no evidence of an association between the exposures and knee replacement (**Figure 4**).

### Sensitivity Analysis

First, the WM method was used for sensitivity analysis. Similar with the results of main IVW analyses, the WM analyses suggested a positive causal relationship between serum DHT (P=0.006) and T (P=4.97×10^−4^) levels and hip OA and the causal effects of serum DHEAS (P=0.017) and T (P=4.10×10^−5^) levels on the risk of hip replacement were statistically significant (**Supplementary Tables 7**–**9**). However, positive associations were found between serum DHT level and overall OA and between serum T level and OA in women, which were inconsistent with our primary findings. Second, in the MR-Egger analysis, nearly all of the intercept terms were centered around the origin, suggesting that there was no horizontal pleiotropy (**Supplementary Tables 7**–**9**). Nevertheless, there were some exceptions such as the P value of the pleiotropy estimate of the relationships between E2 and overall OA and between DHT and overall or hip OA.

The MR-PRESSO analysis was performed to identify outlying SNPs when the number of genetic variants for specific exposure was >3. In the association between serum E_2_ level and overall OA, rs5934505 was identified as an outlier and removed; rs747279 and rs5934505 were also identified as outliers and were excluded from the MR-PRESSO analysis of the association between serum E2 level and knee OA. Rs34790908 was another outlying SNP in the association between serum T level and overall OA. After removing these outliers, the causal association between serum T level and hip OA was still observed in the MR-PRESSO analysis (OR=1.558, 95% CI: 1.193–2.034; P=0.047). Additionally, after removing genetic variants that were directly associated with the outcome measures (rs5934505 for overall OA; rs34790908 for hip OA and hip replacement; and rs4227 for hip OA and hip replacement), the positive causal effects of serum T (hip OA: P=0.001; hip replacement: P=4.52×10^−5^) and DHT (hip OA: P=0.011; hip replacement: P=0.002) levels on risk of hip OA and hip replacement remained significant in the IVW analyses (**Supplementary Table 10**).

## Discussion

This MR study was conducted to investigate the causal relationship between sex steroids levels and OA risk. Our results revealed positive causal effects of serum concentrations of T on the risk of hip OA. There was potential positive association between DHT and hip OA as well while little evidence of the association between sex steroids levels and overall OA was found. Interestingly, males were reported to have higher scores for cartilage damage than age-matched females in a murine OA model induced by destabilization of the medial meniscus (23). Orchiectomized males had less severe damage than their intact counterparts whereas the opposite was true for ovariectomized females, indicating that sex hormones play varied roles in the progression of OA. In fact, the steroid hormones 17β-estradiol, DHEA, and T were shown to promote articular cartilage integration (24).

In a cohort of healthy middle-aged men with no symptoms of knee OA or risk factors, serum free T level was associated with the rate of tibial cartilage loss leading to the development of arthritis 2 years later (25). In a cross-sectional study, a higher concentration of serum T was associated with higher cartilage volume (26), possibly due to greater physical activity and stress on the articular cartilage. Thus, chronic stress and damage to weight-bearing joints—especially the hip and knee—can contribute to OA. However, the protective effects of E2 and DHEAS on OA occurrence are unclear. In a cohort of community-dwelling older subjects, a lower DHEAS was associated with OA irrespective of site and sex (27); and histomorphometric studies in a rabbit model of progressive OA showed that DHEAS treatment reduced cartilage lesions and delayed cartilage degeneration (28, 29). DHEAS was shown to modulate the imbalance between matrix metalloproteinases (MMPs) and its inhibitors (30–33); and intra-articular administration of DHEA reduced aggrecanase expression *in vivo* (34).

In our study, a nominally significant result for the relationship between serum DHT levels and risk of OA in women was found, while little evidence of the sex-specific association between serum T levels and OA was provided. However, a 2-year double-blind cross-sectional analysis of 273 seniors with severe knee OA at an average age of 70.3 years found that a higher T level was associated with less knee disability in non-operated women and less pain (as determined by the Western Ontario and McMaster Universities Osteoarthritis Index) in normal-weight men (35). In another longitudinal investigation on the association between endogenous sex hormone levels and knee OA features and pain, T level was inversely associated with effusion-synovitis volume and pain score in female OA patients (36). However, these findings can only explain the relationship between T level and severity of knee OA symptoms because all of the subjects were OA patients. Besides, our results were based on a non–age-stratified population. A clinical study reported that middle-aged menopausal women with generalized OA had a slightly higher T level and lower circulating SHBG level than control subjects (37).

Unexpectedly, we did not observe any causal association between E2 level and OA risk in women, which may be caused by the male-specific GWAS from which the SNPs for E2 were selected. The marked increase in OA incidence during or soon after menopause is well established. Moreover, estradiol is a known protective factor against OA. The use of oral estrogen was found to be associated with a decreased incidence of radiographic hip OA in elderly Caucasian women (38). A case–control study of women aged ≥45 years found that short-term HRT (up to 5 years) was associated with an increased risk of hip OA, while long-term treatment had a nonsignificant protective effect (39). In premenopausal Caucasian women with knee grade ≥2, a clinical diagnosis of OA was positively correlated with serum estradiol level (40). These findings indicate that the effects of reproductive hormones vary according to age and concentration.

E2 level was shown to be negatively correlated with OA severity and positively correlated with interleukin (IL)-1, IL-6, and tumor necrosis factor (TNF)-α concentrations in the synovial fluid of postmenopausal women (41). In vitro studies have demonstrated that 17β-estradiol treatment enhanced proliferation and viability in chondrocytes by inhibiting mitophagy *via* the G protein-coupled estrogen receptor (GPER/GPR30) and phosphatidylinositol 3-kinase (PI3K)/protein kinase B (AKT) signaling pathways (42, 43), and induced chondrocyte redifferentiation *via* the estrogen receptor (ER)α66/specificity protein (Sp)1/Sp3/SRY-box (SOX)9/p300 protein complex (44). In experiments with mice, a reduction in serum E2 caused by ovariectomy induced severe OA, while supplementary administration of β-estradiol rescued bone turnover and tissue degradation (45). Lower levels of endogenous estrogen and its metabolites were significantly associated with the development of knee OA (46, 47). E2 was shown to inhibit activation of the NOD-, LRR-, and pyrin domain-containing protein (NLRP)3 inflammasome, IL-1β and IL-18 expression, and the catabolic activity of MMPs *via* estrogen receptor or miR-140 (48–52). Our findings suggested that women are more susceptible to OA than men because of differences in sex steroid profiles.

Our results showed a positive causal association between serum T and DHT levels and risk of hip replacement while no relationship was found between E_2_ and arthroplasty due to data deficiencies. A long-term study with a mean follow-up of 12.7 years reported that oral contraceptive use, current HRT use, and longer duration of HRT were associated with increased risk of total knee arthroplasty for OA in women aged 40–69 years (53). However, in another prospective cohort study, the association between incidence of total knee replacement for OA and lower E2 concentration was independent of established risk factors for knee OA, and there was no relationship between E2 and total hip replacement (54). In spite of the exclusion of LD-linkage, rs5934505 for E_2_ and rs727428 for DHT were found to be confounded with T, and rs4227 for T was found to be confounded with DHT by the analysis in PhenoScanner database. Both of them are all located in the same chromosome locus named SHBG. Previous studies suggested that SHBG was significantly associated with T and DHT levels in European ancestry (17) but was not associated with T in Japanese men (55). Moreover, a mutant allele of rs727428 was found to be positively correlated with PCOS in Mediterranean women (56). rs4227 was found to be positively associated with IgA nephropathy in Han Chinese which located in 17p13 7431901 coding MPDU1 gene (57).

Compared to traditional retrospective analyses and case–control prospective studies, MR is less likely to introduce artificial errors and bias. Sex steroid levels fluctuate over time and measurements are often imprecise and experiment design cannot always be perfect. The cost of data collection and analysis can be high, and the MR method can to some extent mitigate data migration; moreover, sequential application of different algorithms in the sensitivity analysis can increase the accuracy and reliability of the results. Nonetheless, our study had several limitations. First, the outcome datasets and representative SNPs were sometimes too limited for us to carry out sensitivity analyses. Second, age-adjusted IVW analyses are necessary to exclude the influence of aging on OA and sex steroid levels, but we did not find any age-stratified OA datasets to perform adjusted IVW analyses. Third, since the GWASs on E2, T and DHT included only male participants in this study, the results of sex-stratified analyses may be influenced by the exposure data. Forth, there might be some dependencies between T and DHT. Finally, our study focused on the causal role of sex steroids in the pathogenesis of OA, but the underlying mechanisms remain to be elucidated.

In conclusion, serum T and DHT levels were causally related to the risk of hip replacement surgery and T was positively associated with risk of hip OA. Further, a nominally significant relationship was found between serum DHT levels and OA in women as well as hip OA. Thus, these sex steroids may contribute to the development of OA. Our findings can guide the development of effective clinical management strategies to maintain joint health and prevent OA, especially in women.

## Data Availability Statement

Summary data used in our study was downloaded from GWAS Catalog (https://www.ebi.ac.uk/gwas/), Neale’s lab (http://www.nealelab.is/uk-biobank) and IEU OpenGWAS project (https://gwas.mrcieu.ac.uk/).

## Ethics Statement

Ethical review and approval was not required for the study on human participants in accordance with the local legislation and institutional requirements. Written informed consent for participation was not required for this study in accordance with the national legislation and the institutional requirements.

## Author Contributions

Y-SY, ZQ, D-QY, WW, SY, H-FH desinged the study. ZQ analyzed the data. Y-SY wrote the manuscript. All authors contributed to the article and approved the submitted version.

## Funding

This work was supported by the National Natural Science Foundation of China (grant no. 81772360).

## Conflict of Interest

The authors declare that the research was conducted in the absence of any commercial or financial relationships that could be construed as a potential conflict of interest.
