# Supplementary material for: Sex Steroids and Osteoarthritis: A Mendelian Randomization Study
Source: Front Endocrinol (Lausanne). 2021 Jun 23;12:683226. doi: 10.3389/fendo.2021.683226 (PMC8261132; doi:10.3389/fendo.2021.683226)
Supplement: Supplementary file 1 [file Table_1.docx]

Supplementary Material

Supplemental Table 1. Calculation of linkage disequilibrium of the selected SNPs

E_2_-associated SNPs

| RS number | rs727479 | rs16964258 |
| --- | --- | --- |
| rs727479 | 1.0 | 0.011 |
| rs16964258 | 0.011 | 1.0 |

DHEAS-associated SNPs

| RS number | rs740160 | rs17277546 |
| --- | --- | --- |
| rs740160 | 1.0 | 0.0 |
| rs17277546 | 0.0 | 1.0 |

| RS number | rs2497306 | rs2185570 |
| --- | --- | --- |
| rs2497306 | 1.0 | 0.009 |
| rs2185570 | 0.009 | 1.0 |

T-associated SNPs

| RS number | rs4239258 | rs34790908 | rs727428 |
| --- | --- | --- | --- |
| rs4239258 | 1.0 | 0.014 | 0.055 |
| rs34790908 | 0.014 | 1.0 | 0.194 |
| rs727428 | 0.055 | 0.194 | 1.0 |

DHT-associated SNPs

| RS number | rs4151121 | rs4265880 | rs4227 |
| --- | --- | --- | --- |
| rs4151121 | 1.0 | 0.012 | 0.176 |
| rs4265880 | 0.012 | 1.0 | 0.015 |
| rs4227 | 0.176 | 0.015 | 1.0 |

The included SNPs in each table are on the same chromosome.

Population = (CEU) Utah Residents from North and West Europe.

Supplementary Table 2. Potential confounders associated with selected SNPs.

| Exposure | SNP | Trait | P value |
| --- | --- | --- | --- |
| E_2_ | rs727479 | Heel bone mineral density | 2.44E-22 |
|  | rs16964258 | / | / |
|  | rs5934505 | Androgen levels | 2.00E-08 |
|  |  | Testosterone | 2.83E-10 |
|  | rs5951794 | / | / |
| DHEAS | rs6738028 | Lymphocyte count | 2.61E-10 |
|  |  | Mean corpuscular hemoglobin | 1.56E-11 |
|  | rs740160 | / | / |
|  | rs17277546 | Metabolic traits | 9.00E-40 |
|  | rs2497306 | Eosinophil count | 2.64E-15 |
|  |  | Type II diabetes | 1.50E-12 |
|  | rs2185570 | / | / |
|  | rs7181230 | Systolic blood pressure | 1.43E-09 |
|  | rs2637125 | Cholelithiasis | 3.90E-09 |
| T | rs10822186 | Granulocyte count | 6.16E-20 |
|  |  | Neutrophil count | 3.75E-21 |
|  |  | Platelet count | 1.42E-61 |
|  |  | White blood cell count | 6.12E-20 |
|  |  | Triglycerides | 1.39E-14 |
|  |  | Diastolic blood pressure | 7.77E-11 |
|  |  | Self-reported cholelithiasis or gall stones | 3.70E-08 |
|  | rs4239258 | / | / |
|  | rs34790908 | Systolic blood pressure | 1.25E-08 |
|  | rs727428 | Circulating sex hormone binding globulin levels | 1.31E-16 |
|  |  | Serum dihydrotestosterone DHT level | 1.47E-11 |
| DHT | rs4151121 | Testosterone | 1.63E-09 |
|  | rs4265880 | / | / |
|  | rs4227 | Circulating sex hormone binding globulin levels | 1.67E-11 |
|  |  | Serum testosterone T level | 3.99E-08 |
|  |  | IgA nephropathy | 4.00E-10 |
|  |  | Systolic blood pressure | 2.70E-08 |
|  |  | Testosterone | 3.81E-16 |

Supplemental Table 3. The genetic associations between the selected SNPs and overall, hip and knee OA

|  | SNP | EA | Overall OA | | Hip OA | | Knee OA | |
| --- | --- | --- | --- | --- | --- | --- | --- | --- |
| Exposure |  |  | β(SE) | P value | β(SE) | P value | β(SE) | P value |
| E_2_ | rs727479 | A | -0.011 (0.006) | 0.067 | 0.006 (0.012) | 0.604 | -0.031 (0.010) | 0.002 |
| E_2_ | rs16964258 | G | -0.006 (0.013) | 0.631 | -0.015 (0.028) | 0.601 | -0.004 (0.023) | 0.867 |
| E_2_ | rs5934505 | C | 0.022 (0.005) | 3.12E-05 | 0.032 (0.012) | 0.007 | 0.020 (0.009) | 0.028 |
| E_2_ | rs5951794 | G | 0.007 (0.005) | 0.194 | -0.001 (0.011) | 0.967 | 0.013 (0.009) | 0.127 |
| DHEAS | rs6738028 | G | 0.001 (0.006) | 0.805 | -0.011 (0.012) | 0.345 | 0.005 (0.010) | 0.612 |
| DHEAS | rs740160 | T | 0.007 (0.013) | 0.591 | 0.056 (0.028) | 0.044 | 0.002 (0.022) | 0.927 |
| DHEAS | rs17277546 | A | -0.018 (0.013) | 0.173 | 0.000 (0.028) | 0.993 | -0.006 (0.022) | 0.769 |
| DHEAS | rs2497306 | C | 0.007 (0.006) | 0.226 | 0.002 (0.012) | 0.843 | 0.013 (0.009) | 0.171 |
| DHEAS | rs2185570 | C | 0.003 (0.008) | 0.682 | 0.006 (0.017) | 0.741 | -0.008 (0.014) | 0.538 |
| DHEAS | rs7181230 | G | 0.007 (0.006) | 0.223 | 0.009 (0.012) | 0.473 | 0.013 (0.010) | 0.199 |
| DHEAS | rs2637125 | A | 0.014 (0.008) | 0.071 | 0.030 (0.016) | 0.062 | -0.016 (0.013) | 0.207 |
| T | rs10822186 | A | -0.009 (0.006) | 0.105 | -0.033 (0.012) | 0.004 | -0.003 (0.009) | 0.761 |
| T | rs4239258 | A | -0.002 (0.014) | 0.869 | -0.024 (0.030) | 0.438 | 0.023 (0.024) | 0.336 |
| T | rs34790908 | T | 0.022 (0.006) | 4.50E-04 | 0.055 (0.013) | 3.09E-05 | 0.013 (0.010) | 0.226 |
| T | rs727428 | T | 0.005 (0.006) | 0.377 | -0.023 (0.012) | 0.048 | 0.009 (0.009) | 0.331 |
| DHT | rs4151121 | G | 0.013 (0.006) | 0.022 | 0.034 (0.012) | 0.005 | 0.008 (0.010) | 0.381 |
| DHT | rs4265880 | T | -0.001 (0.014) | 0.938 | -0.022 (0.030) | 0.470 | 0.025 (0.024) | 0.310 |
| DHT | rs4227 | G | 0.020 (0.006) | 0.001 | 0.056 (0.013) | 1.58E-05 | 0.013 (0.010) | 0.209 |

EA = effect allele; β = per allele effect on the outcome; SE = standard error; P value = p-value for the genetic association.

Supplemental Table 4. The genetic associations between the selected SNPs and OA in women and men

|  | SNP | EA | OA in women | | OA in men | |
| --- | --- | --- | --- | --- | --- | --- |
| Exposure |  |  | β(SE) | P value | β(SE) | P value |
| E_2_ | rs727479 | A | -1.61E-03 (9.93E-04) | 0.105 | -9.61E-04 (8.80E-04) | 0.275 |
| E_2_ | rs16964258 | G | -2.99E-03 (2.30E-03) | 0.195 | -1.90E-03 (2.04E-03) | 0.352 |
| E_2_ | rs5934505 | C | 5.78E-04 (1.07E-03) | 0.589 | 3.65E-04 (6.70E-04) | 0.586 |
| E_2_ | rs5951794 | G | 1.29E-04 (1.02E-03) | 0.899 | -4.36E-04 (6.37E-04) | 0.494 |
| DHEAS | rs6738028 | G | -2.19E-04 (9.72E-04) | 0.821 | 4.82E-04 (8.62E-04) | 0.576 |
| DHEAS | rs740160 | T | -1.48E-04 (2.26E-03) | 0.948 | 1.67E-03 (1.99E-03) | 0.403 |
| DHEAS | rs17277546 | A | 8.33E-04 (2.23E-03) | 0.709 | -1.89E-03 (1.96E-03) | 0.335 |
| DHEAS | rs2497306 | C | 1.30E-03 (9.46E-04) | 0.168 | -2.20E-04 (8.41E-04) | 0.793 |
| DHEAS | rs2185570 | C | 2.77E-03 (1.38E-03) | 0.045 | 4.70E-04 (1.22E-03) | 0.701 |
| DHEAS | rs7181230 | G | 1.52E-03 (9.95E-04) | 0.128 | 6.59E-04 (8.81E-04) | 0.454 |
| DHEAS | rs2637125 | A | 3.92E-03 (1.30E-03) | 0.003 | -4.29E-04 (1.15E-03) | 0.710 |
| T | rs10822186 | A | -1.36E-03 (9.50E-04) | 0.153 | -2.86E-04 (8.42E-04) | 0.734 |
| T | rs4239258 | A | -3.48E-03 (2.49E-03) | 0.162 | -2.15E-03 (2.21E-03) | 0.329 |
| T | rs34790908 | T | 1.74E-03 (1.06E-03) | 0.102 | -6.71E-04 (9.44E-04) | 0.477 |
| T | rs727428 | T | 3.11E-04 (9.53E-04) | 0.744 | 6.16E-04 (8.46E-04) | 0.467 |
| DHT | rs4151121 | G | 1.26E-03 (9.82E-04) | 0.200 | 1.28E-04 (8.70E-04) | 0.883 |
| DHT | rs4265880 | T | -3.42E-03 (2.48E-03) | 0.168 | -2.16E-03 (2.20E-03) | 0.327 |
| DHT | rs4227 | G | 8.10E-04 (1.06E-03) | 0.444 | -1.52E-05 (9.38E-04) | 0.987 |

EA = effect allele; β = per allele effect on the outcome; SE = standard error; P value = p-value for the genetic association.

Supplemental Table 5. The genetic associations between the selected SNPs and joint replacement

|  | SNP | EA | Hip replacement | | Knee replacement | |
| --- | --- | --- | --- | --- | --- | --- |
| Exposure |  |  | β(SE) | P value | β(SE) | P value |
| DHEAS | rs6738028 | G | -1.80E-04 (2.66E-04) | 0.500 | -1.53E-05 (2.34E-04) | 0.950 |
| DHEAS | rs2497306 | C | 2.83E-04 (2.59E-04) | 0.280 | -1.51E-04 (2.28E-04) | 0.510 |
| DHEAS | rs2185570 | C | 5.81E-04 (3.79E-04) | 0.130 | -2.92E-04 (3.33E-04) | 0.380 |
| DHEAS | rs7181230 | G | 3.24E-04 (2.73E-04) | 0.230 | 3.93E-04 (2.40E-04) | 0.100 |
| DHEAS | rs2637125 | A | 8.17E-04 (3.55E-04) | 0.022 | 9.87E-05 (3.13E-04) | 0.750 |
| T | rs10822186 | A | -7.65E-04 (2.60E-04) | 0.003 | 6.10E-05 (2.29E-04) | 0.790 |
| T | rs34790908 | T | 1.16E-03 (2.92E-04) | 7.10E-05 | 1.17E-04 (2.57E-04) | 0.650 |
| T | rs727428 | T | -7.50E-04 (2.61E-04) | 0.004 | -6.67E-05 (2.30E-04) | 0.770 |
| DHT | rs4151121 | G | 8.53E-04 (2.69E-04) | 0.001 | 8.10E-05 (2.37E-04) | 0.730 |
| DHT | rs4227 | G | 1.18E-03 (2.91E-04) | 4.90E-05 | 1.95E-04 (2.56E-04) | 0.450 |

EA = effect allele; β = per allele effect on the outcome; SE = standard error; P value = p-value for the genetic association.

Supplemental Table 6. Exposure-related SNPs, original articles and ethical approval were listed as followed.

| Exposure |  | Original article | Relevant ethical approval |
| --- | --- | --- | --- |
| Estrogen level | 11097 European men | Genetic Determinants of Circulating Estrogen Levels and Evidence of a Causal Effect of Estradiol on Bone Density in Men | FHS (5R01DK092938-04, 1R01AG31206-01),  GOOD study (HEALTH-F2-2008-201865-GEFOS), the Invecchiare in Chianti study (ICS110.1/RF97.71),  LURIC study (201668, 305739),  the Multi-Ethnic Study of Atherosclerosis study (HL074406, HL074338),  MrOS Sweden, the MrOS Sweden Malmö study, the MrOS US study (U01 AG027810, U01 AG042124, U01 AG042139, U01 AG042140, U01 AG042143, U01 AG042145, U01 AG042168, U01 AR066160, UL1 TR000128, and RC2 AR058973) and RS1. |
| T and DHT levels | 3225 European descent | Genome-wide association study identifies a new locus JMJD1C at 10q21 that may influence serum androgen levels in men | REDUCE study supported by a national cancer institute RC2 grant (ca148463). |
| DHEAS level | 14846 European individuals | Eight Common Genetic Variants Associated with Serum DHEAS Levels Suggest a Key Role in Ageing Mechanisms | TwinsUK, Framingham Heart Study (FHS), SHIP, Rotterdam Study (RS1), InCHIANTI, Health ABC, and GOOD. |

Supplemental Table 7. Weighted median, MR-Egger and MR-PRESSO analysis for genetic associations between exposures and overall, hip and knee OA

| Exposure | Method | Overall OA | | Hip OA | | Knee OA | |
| --- | --- | --- | --- | --- | --- | --- | --- |
|  |  | Effect (95% CI) | P value | Effect (95% CI) | P value | Effect (95% CI) | P value |
| E_2_ | Weighted median | 0.996 (0.988, 1.003) | 0.265 | 1.003 (0.988, 1.017) | 0.725 | 0.992 (0.978, 1.006) | 0.271 |
| E_2_ | MR-Egger Estimate | 0.976 (0.951, 1.002) | 0.073 | 0.984 (0.943, 1.026) | 0.449 | 0.961 (0.921, 1.003) | 0.067 |
| E_2_ | MR-Egger Intercept | 1.030 (1.002, 1.058) | 0.036 | 1.027 (0.982, 1.074) | 0.244 | 1.041 (0.996, 1.088) | 0.076 |
| E_2_ | MR-PRESSO | 0.997 (0.988, 1.006) | 0.544 | 1.007 (0.989, 1.025) | 0.525 | 1.007 (0.987, 1.028) | 0.627 |
| DHEAS | Weighted median | 0.978 (0.871, 1.097) | 0.702 | 1.005 (0.777, 1.301) | 0.968 | 1.086 (0.911, 1.296) | 0.358 |
| DHEAS | MR-Egger Estimate | 1.046 (0.821, 1.331) | 0.717 | 1.058 (0.619, 1.808) | 0.837 | 1.201 (0.872, 1.653) | 0.262 |
| DHEAS | MR-Egger Intercept | 0.996 (0.981, 1.012) | 0.612 | 0.999 (0.965, 1.034) | 0.935 | 0.991 (0.971, 1.012) | 0.408 |
| DHEAS | MR-PRESSO | 0.988 (0.897, 1.089) | 0.818 | 1.037 (0.840, 1.279) | 0.747 | 1.063 (0.937, 1.206) | 0.381 |
| T | Weighted median | 1.054 (0.938, 1.185) | 0.373 | 1.527 (1.203, 1.938) | 4.97E-04 | 0.948 (0.799, 1.126) | 0.544 |
| T | MR-Egger Estimate | 0.910 (0.460, 1.800) | 0.787 | 0.881 (0.390, 1.989) | 0.760 | 0.754 (0.440, 1.293) | 0.305 |
| T | MR-Egger Intercept | 1.014 (0.964, 1.067) | 0.586 | 1.045 (0.984, 1.110) | 0.153 | 1.020 (0.980, 1.062) | 0.322 |
| T | MR-PRESSO | 1.018 (0.897, 1.155) | 0.808 | 1.558 (1.193, 2.034) | 0.047 | 0.979 (0.842, 1.139) | 0.804 |
| DHT | Weighted median | 1.132 (1.021, 1.256) | 0.019 | 1.394 (1.100, 1.765) | 0.006 | 1.073 (0.920, 1.252) | 0.369 |
| DHT | MR-Egger Estimate | 0.895 (0.728, 1.101) | 0.295 | 0.842 (0.499, 1.421) | 0.519 | 0.779 (0.551, 1.102) | 0.159 |
| DHT | MR-Egger Intercept | 1.027 (1.003, 1.051) | 0.025 | 1.063 (1.002, 1.128) | 0.043 | 1.035 (0.995, 1.076) | 0.087 |

For MR-PRESSO analysis on the association between E2 and overall OA, 3 SNPs were included since rs5934505 was identified as an outlier and was removed. For MR-PRESSO analysis on the association between E2 and knee OA, rs727479 and rs5934505 were identified as outliers and were removed from instrumental variables. And for MR-PRESSO analysis on the association between T and overall OA, rs34790908 was identified as an outlier and was removed from instrumental variables. CI = confidence interval; P value = p-value of the causal estimate.

Supplemental Table 8. Weighted median, MR-Egger and MR-PRESSO analysis for genetic associations between exposures and OA in women and men

| Exposure | Method | OA in women | | OA in men | |
| --- | --- | --- | --- | --- | --- |
|  |  | Effect (95% CI) | P value | Effect (95% CI) | P value |
| E_2_ | Weighted median | 0.999 (0.998, 1.000) | 0.059 | 0.999 (0.998, 1.000) | 0.172 |
| E_2_ | MR-Egger Estimate | 0.997 (0.995, 1.000) | 0.055 | 0.999 (0.997, 1.001) | 0.224 |
| E_2_ | MR-Egger Intercept | 1.002 (0.999, 1.005) | 0.162 | 1.001 (0.999, 1.003) | 0.422 |
| E_2_ | MR-PRESSO | 0.999 (0.998, 1.000) | 0.152 | 0.999 (0.999, 1.000) | 0.173 |
| DHEAS | Weighted median | 0.993 (0.971, 1.015) | 0.506 | 1.006 (0.991, 1.022) | 0.406 |
| DHEAS | MR-Egger Estimate | 0.978 (0.928, 1.032) | 0.419 | 1.016 (0.987, 1.046) | 0.283 |
| DHEAS | MR-Egger Intercept | 1.000 (0.997, 1.004) | 0.778 | 0.999 (0.997, 1.001) | 0.472 |
| DHEAS | MR-PRESSO | 0.985 (0.965, 1.006) | 0.209 | 1.006 (0.999, 1.013) | 0.128 |
| T | Weighted median | 1.023 (1.004, 1.042) | 0.016 | 0.996 (0.981, 1.012) | 0.640 |
| T | MR-Egger Estimate | 1.019 (0.955, 1.086) | 0.573 | 1.018 (0.972, 1.066) | 0.443 |
| T | MR-Egger Intercept | 1.000 (0.995, 1.004) | 0.898 | 0.999 (0.995, 1.002) | 0.400 |
| T | MR-PRESSO | 1.015 (1.000, 1.030) | 0.152 | 0.999 (0.988, 1.010) | 0.871 |
| DHT | Weighted median | 1.013 (0.999, 1.028) | 0.072 | 1.002 (0.989, 1.015) | 0.770 |
| DHT | MR-Egger Estimate | 1.017 (0.981, 1.054) | 0.352 | 1.015 (0.983, 1.048) | 0.358 |
| DHT | MR-Egger Intercept | 0.999 (0.995, 1.003) | 0.784 | 0.999 (0.995, 1.002) | 0.458 |

CI = confidence interval; P value = p-value of the causal estimate.

Supplemental Table 9. Weighted median, MR-Egger and MR-PRESSO analysis for genetic associations between exposures and joint replacement

| Exposure | Method | Hip replacement | | Knee replacement | |
| --- | --- | --- | --- | --- | --- |
|  |  | Effect (95% CI) | P value | Effect (95% CI) | P value |
| DHEAS | Weighted median | 0.992 (0.986, 0.999) | 0.017 | 1.002 (0.997, 1.007) | 0.514 |
| DHEAS | MR-Egger Estimate | 0.983 (0.964, 1.003) | 0.104 | 0.997 (0.983, 1.010) | 0.650 |
| DHEAS | MR-Egger Intercept | 1.001 (1.000, 1.002) | 0.193 | 1.000 (1.000, 1.001) | 0.396 |
| DHEAS | MR-PRESSO | 0.996 (0.989, 1.003) | 0.312 | 1.002 (0.999, 1.006) | 0.234 |
| T | Weighted median | 1.013 (1.007, 1.019) | 4.10E-05 | 1.001 (0.996, 1.006) | 0.685 |
| T | MR-Egger Estimate | 1.008 (0.957, 1.061) | 0.776 | 1.010 (0.970, 1.052) | 0.624 |
| T | MR-Egger Intercept | 1.000 (0.997, 1.004) | 0.832 | 0.999 (0.997, 1.002) | 0.644 |

CI = confidence interval; P value = p-value of the causal estimate.

Supplemental Table 10. IVW analysis with the removal of SNPs directly associated with the included outcomes

| Exposure | Outcome |  | Hip replacement | |
| --- | --- | --- | --- | --- |
|  |  | Removed SNP | Effect (95% CI) | P value |
| E_2_ | Overall OA | rs5934505 | 0.997 (0.991, 1.003) | 0.293 |
| T | Hip OA | rs34790908 | 1.401 (1.140, 1.721) | 0.001 |
| T | Hip replacement | rs34790908 | 1.011 (1.006, 1.017) | 4.52E-05 |
| DHT | Hip OA | rs4227 | 1.253 (1.052, 1.493) | 0.011 |
| DHT | Hip replacement | rs4227 | 1.009 (1.003, 1.014) | 0.002 |

CI = confidence interval; P value = p-value of the causal estimate.
